# Performance in decoding and writing of children with Developmental Language Disorder: preliminary data

**DOI:** 10.1590/2317-1782/20232022318en

**Published:** 2023-10-23

**Authors:** Aparecido José Couto Soares, Gabriele Hilário Cardoso Santos, Débora Maria Befi-Lopes

**Affiliations:** 1 Departamento de Fonoaudiologia da Universidade Federal de São Paulo - UNIFESP - São Paulo (SP), Brasil.; 2 Departamento de Fisioterapia, Fonoaudiologia e Terapia Ocupacional, Faculdade de Medicina, Universidade de São Paulo - USP - São Paulo (SP), Brasil.

**Keywords:** Language, Specific Language Disorder, Reading, Learning, Evaluation

## Abstract

**Purpose:**

to verify the performance of children with Developmental Language Disorder in decoding and writing tests in order to better understand their manifestations and the process of acquiring written language skills.

**Methods:**

The study subjects were 80 children. The Research Group consisted of 16 children diagnosed with Developmental Language Disorder, 13 males and 3 females, mean age of 7.3. The Control Group counted on 64 subjects paired in gender, age, education and socioeconomic level with the Control Group in a 4:1 ratio. The ability to decode words and pseudowords of both groups was evaluated, measuring the time spent to correctly read words and the percentage of correct answers, also considering the length of the word/pseudoword. The writing evaluation was carried out in the control group, which had its spelling errors analyzed and categorized. All data underwent descriptive and inferential statistical analysis.

**Results:**

The data indicated a longer decoding time and a lower percentage of correct answers for the children from the Research Group. Regarding spelling errors, there was a predominance of arbitrary spelling errors.

**Conclusion:**

The data showed that children with Developmental Language Disorder tend to have a longer decoding time, greater percentage of errors than their peers and tend to present spelling errors more concentrated in natural orthography.

## INTRODUCTION

Developmental Language Disorder (DLD) is characterized by the presence of significant disorders in the language acquisition and development process, excluding children with alterations justified by socioenvironmental factors, bi or multilingualism, and biomedical conditions where language alterations are expected^(1,2)^.

It is commonly observed impairments in lexical acquisition in these children, which varies according to the degree of the disorder, and the child may have difficulties in expressive and receptive vocabulary. Also, impairment in phonological aspects is an important feature, and these children often have unintelligible speech. Some studies^(3,4)^ point to important deficits in the components of phonological processing in children with DLD, such as phonological short-term memory and phonological awareness, which are considered fundamental skills for the process of language acquisition in its written form^(3)^.

In this context, we know that phonological processing skills are associated with success in the learning process because together, they are responsible for the ability to analyze the sound structure of speech, retention of information, and quick access to representations of the phonological information of the language^(5)^. However, these are impaired abilities in DLD children due to alterations in several language subsystems^(1)^.

Therefore, these disorders lead to a longer time for linguistic solidification in children with DLD than in people in typical development and lead to important repercussions in the process of acquisition of written language^(4)^. These children have difficulties related to reading and writing, probably resulting from the impairment of oral language and phonological processing characteristic of DLD^(6)^. There is a growing number of studies related to the written language skills of children with DLD, as well as the importance of their characterization for making differential diagnoses in Specific Learning Disorders and even the co-occurrence of these conditions^(7)^.

However, there are few Brazilian studies dedicated to better understanding these children's written language disorders, as well as their acquisition process. This is because, until the paradigm and nomenclature changed from Specific Language Disorder (SLD) to DLD(1), manifestations of written language were little investigated in this population, mainly in Brazil. However, the importance of a holistic approach to the alterations of DLD children is evident, especially considering that this disorder longitudinally affects all language subsystems^(4)^, which will lead to different impacts throughout the individual's life and, consequently, in their school career.

Thus, studies that investigate manifestations in the written language of children with DLD are of paramount importance to better understand the impairments of this population and outline not only therapeutic plans involving written language but also to develop actions that promote the development of educational public policies for these children who are not currently supported by any legislation. As hypotheses, it is expected that children with DLD present impairments in the decoding and acquisition of writing.

Therefore, this study aimed to verify the performance of children diagnosed with DLD in decoding and writing tests under dictation.

## METHODS

Retrospective cross-sectional study, approved by the Ethics and Research Committee of the School Medicine of the University of São Paulo under nº 2,262,300. The study was conducted in the Laboratory for Speech-Language Investigation in Pediatrics of the Speech-Language Pathology graduation degree of the University of São Paulo. Because the study was retrospective, carried out in a database, the Informed Consent Form (ICF) was waived.

The participants in this study were 80 children aged 6 to 10 years old, divided into two groups. The Research Group (RG) was composed of 16 children, 13 males, and 3 females, with a mean age of 7.3, with a diagnosis of DLD based on recent international criteria^(1,2)^, who were treated at a speech-language therapy school clinic. The people in the service are primarily of medium-low socioeconomic level. It is important to point out that this variable was considered when evaluating and diagnosing DLD, according to the most recent guidelines, which include an evaluation battery of all language subsystems and their underlying abilities^(1,2)^. The inclusion criteria for this group were: having a diagnosis of DLD; being of formal school age (6 to 10 years old); and being regularly enrolled in Elementary School. The Control Group (CG) had 64 subjects paired in gender, age, education, and socioeconomic level with the RG in a 4:1 ratio, that is, each child in the RG was paired with 4 children in the CG.

The CG was built specifically for the decoding test and has children with typical oral language development, reading, and writing, confirmed by speech-language therapy procedures performed in a previous study^(8)^. The mean age of the CG was 7.2. The inclusion criteria for the CG were: not having complaints or alterations in oral and written language; being regularly enrolled in elementary school; not having learning complaints; having adequate performance in speech-language screening performed in the previous study^(8)^. RG participants were assessed for their decoding and writing skills and CG children only for decoding. This is because the decoding test used has specific parameters and variables that were recently published^(8)^ and the writing analysis is performed by an instrument published in the form of a standardized test^(9)^, eliminating the need for a CG for such variable.

For decoding evaluation and analysis, the Decoding Development Monitoring Protocol (PRADE- *Protocolo de Acompanhamento do Desenvolvimento da Decodificação*)^(8)^ was used and it consists of linguistically balanced words according to the Brazilian Portuguese (BP) decoding rules, also respecting the variation in word length from mono to polysyllables for children in this school age group. The test also has non-words that were derived from real words and that also follow the BP decoding rules, as well as the variation from mono to polysyllables. Both tasks were carried out face-to-face and consisted of asking the child to decode the words in the way they believed to be the correct way. The words are presented starting with monosyllables, followed by disyllables, and so on. When the child makes ten consecutive errors the test is finished. The procedure is the same for words and non-words. The correct word decoding time and the percentage of correct answers were counted both for each type of stimulus (from mono to polysyllable) and for total values for the category (words or non-words). The choice of such an instrument was because it specifically analyzes decoding, which is known to be a fundamental skill for the later stages of literacy, and because it contains stimuli that are based on the structure of Brazilian Portuguese, with adequate linguistic balance, as described above. Data were tabulated in a specific spreadsheet and underwent statistical analysis.

Regarding writing, a list of words used in a previous study was used, which proved to be adequate for children with DLD^(10).^ The test consists of an eight-word dictation and eight pseudowords; both word lists were composed of two-syllable words with CV (consonant-vowel) structure; in addition, the words have phonographemic correspondence considered transparent. An analysis of the writing performance was carried out and, for children with alphabetic writing, the categorization of the spelling errors profile was performed based on the guidelines of the Pro-Orthography Test^(9)^. In this perspective, the errors were classified, in percentage, in natural and arbitrary spelling errors^(9)^. Data were tabulated in a specific spreadsheet and underwent statistical analysis.

Statistical analysis was performed to characterize the groups in reading time and percentage of correct answers according to the type and length of words, in addition to the total values; the effect of the group, the type of word, and the length of the word on the time and percentage of correct answers were also investigated. The statistical significance value adopted was equal to 5% (*p* ≤ 0.05). The SPSS Statistics software, version 28.0, was used. Generalized Estimation Equations (GEE) were also carried out to verify the effects for each variable separately, within the group, and also the effects of the interaction between all the studied variables (type of word, length, and total values) between the groups. Regarding writing, the percentage of children with an alphabetic level of writing was verified, and, of these, the percentage of types of errors, natural or arbitrary spelling.

## RESULTS

In the analysis of the writing of the children classified at the alphabetic level, the data indicated that, on average, 76.58% of the errors were natural spelling and 35.13% were arbitrary spelling. There was also great variability in the standard deviation with values of 22.95 and 18.68, respectively.

Regarding decoding, a longer decoding time was observed in the RG compared to the CG (Table 1). Furthermore, word length was an important variable and led to increased reading time in both groups, but to a greater extent in RG. This fact was observed both in the decoding of words and pseudowords (Table 1). Regarding the percentage of correct answers for both words and pseudowords, the performance of the RG was considerably lower when compared to the CG (Table 1).

**Table 1:** Descriptive values of reading time (in seconds) and percentage of correct answers according to word type, word length, and group

**READING TIME**										
**Word type**	**Word extension**	**Group**	**n**	**Mean**	**SD**	**CI 95%**		**Median**	**Min.**	**Max.**
						**LL**	**UL**			
Word	Monosyllable	CG	54	7.09	2.56	6.43	7.72	7.10	1.20	14.70
		RG	16	19.56	7.59	16.50	23.12	18.00	11.00	37.00
	Disyllable	CG	52	23.86	10.11	21.28	26.54	22.10	1.10	51.30
		RG	14	72.71	42.67	55.70	95.00	66.00	29.00	201.00
	Trisyllable	CG	50	32.26	14.42	28.17	36.39	32.45	5.50	66.20
		RG	9	109.89	36.08	88.00	132.00	106.00	61.00	183.00
	Polysyllable (4 syllables)	CG	42	38.69	24.56	32.19	46.87	32.15	5.20	137.40
		RG	6	93.50	22.42	76.33	111.20	98.50	64.00	118.00
	Polysyllable (5 syllables)	CG	41	22.00	8.59	19.55	24.66	20.10	5.70	39.50
		RG	5	58.00	22.52	43.40	72.60	57.00	39.00	94.00
	Total	CG	55	104.80	52.25	90.58	119.42	113.00	4.50	209.00
		RG	16	198.19	123.19	142.29	253.37	223.00	18.00	408.00
Pseudoword	Monosyllable	CG	45	8.93	2.85	8.23	9.64	8.50	2.10	19.50
		RG	9	22.78	12.47	16.67	30.44	17.00	12.00	52.00
	Disyllable	CG	42	30.58	9.15	28.05	33.12	28.20	17.80	57.20
		RG	8	94.00	44.04	64.63	124.62	84.50	45.00	154.00
	Trisyllable	CG	45	42.28	14.88	37.82	46.16	41.80	7.60	85.30
		RG	6	129.67	34.94	105.33	157.54	138.50	88.00	179.00
	Polysyllable (4 syllables)	CG	42	37.97	15.13	33.62	42.94	36.70	8.20	79.20
		RG	4	114.25	38.35	86.23	146.25	114.50	68.00	160.00
	Polysyllable (5 syllables)	CG	39	24.42	9.15	21.75	27.02	24.20	6.80	46.20
		RG	4	65.75	17.21	54.25	78.56	63.00	49.00	88.00
	Total	CG	48	127.84	53.91	110.48	142.97	133.60	6.50	219.20
		RG	9	272.56	153.87	180.79	362.44	273.00	12.00	553.00
**PERCENTAGE OF CORRECT ANSWERS**										
**Word type**	**Word extension**	**Group**	**n**	**Mean**	**SD**	**CI 95%**		**Median**	**Min.**	**Max.**
						**LL**	**UL**			
Word	Monosyllable	CG	64	64.06	41.81	54.43	73.78	83.33	0.00	100
		RG	16	56.56	28.03	42.80	69.48	52.50	16.00	100
	Disyllable	CG	64	55.18	40.24	45.63	64.82	75.00	0.00	100
		RG	16	49.26	31.47	33.02	64.52	49.98	0.00	93.75
	Trisyllable	CG	64	52.41	41.41	42.07	63.14	59.09	0.00	100
		RG	16	25.52	30.55	12.15	39.98	14.28	0.00	85.71
	Polysyllable (4 syllables)	CG	64	50.10	42.12	39.85	60.54	62.50	0.00	100
		RG	16	16.80	28.93	3.91	32.03	0.00	0.00	81.25
	Polysyllable (5 syllables)	CG	64	51.76	43.58	41.80	61.52	68.75	0.00	100
		RG	16	17.19	29.18	5.47	31.02	0.00	0.00	87.50
	Total	CG	64	51.94	39.81	42.55	61.73	65.71	0.00	97.14
		RG	16	25.37	30.45	10.72	40.61	13.50	0.00	86.19
Pseudoword	Monosyllable	CG	64	51.04	37.66	41.94	60.16	66.67	0.00	100
		RG	16	39.79	39.07	21.87	57.29	33.33	0.00	100
	Disyllable	CG	64	51.56	40.72	42.29	60.84	62.50	0.00	100
		RG	16	30.98	34.02	15.82	46.61	19.75	0.00	100
	Trisyllable	CG	64	43.11	33.94	35.31	51.12	54.55	0.00	95.45
		RG	16	21.87	32.69	6.53	36.63	0.00	0.00	86.36
	Polysyllable (4 syllables)	CG	64	33.30	29.86	25.98	40.71	37.50	0.00	87.50
		RG	16	16.02	32.19	1.17	32.42	0.00	0.00	93.75
	Polysyllable (5 syllables)	CG	64	36.52	35.17	28.91	44.73	37.50	0.00	100
		RG	16	12.50	27.76	1.56	25.00	0.00	0.00	87.50
	Total	CG	64	41.50	32.89	34.05	49.27	52.14	0.00	92.86
		RG	16	23.09	29.36	10.14	37.00	13.54	0.00	88.52

Caption: n = number of participants; SD = Standard deviation; Min. = Minimum; Max. = Maximum; CI 95% = 95% confidence interval calculated with 1000 bootstrap samples; LL = lower limit; UL = upper limit; CG = Control Group; RG = Research Group

Table 2 confirms the analyzed points regarding joint decoding, indicating a statistical difference in the multiple variables of this research. Thus, we observed interaction between all variables, that is, they all influence the decoding performance; however, the greatest impact was observed in the RG, with losses being observed in all the studied variables (word/pseudoword, word length/pseudoword, total values of decoding time and percentage of correct answers).

**Table 2:** Test of effects for each variable and for the interaction between the variables of the Generalized Estimation Equations (GEE) prepared for time and percentage of correct answers

**Variables**	**Par.**		**Effects**						
		**intercept**	**G**	**T**	**L**	**G x T**	**G x L**	**T x L**	**G x T x L**
Time	Wald's X^2^	10396.960	159.466	29.301	791.129	2.357	10.365	5.758	6.574
	gl	1	1	1	4	1	4	4	4
	p	< 0.001*	**< 0.001** *	< 0.001*	< 0.001*	0.125	0.035*	0.218	0.160
Hits (%)	Wald's X^2^	269.657	4.320	14.827	26.053	2.880	11.609	2.076	1.229
	gl	1	1	1	4	1	4	4	4
	p	< 0.001*	0.038*	< 0.001*	< 0.001*	0.090	0.021*	0.722	0.873

Wald's X^2^ test

* Statistically significant value at the 5% level (*p* ≤ 0.05)

Caption: Par = Parameter; gl = degrees of freedom; G = Group; T = Type of word; L = Word length

## DISCUSSION

This study aimed to verify the performance of children diagnosed with DLD in decoding and writing tests to better understand their manifestations and the process of acquiring written language skills in this population. The basis for the acquisition of written language comes from skills acquired and improved from oral language^(11)^, which occurs more slowly in children with developmental language disorder^(4)^.

In this context, considering the children at the alphabetic writing level, a higher percentage of natural than arbitrary writing errors was noted, which corroborates the hypothesis that the alterations in the phonological processing of these children hinder the phoneme-grapheme conversion and, consequently, the development of writing^(11)^.

It is important to point out that in recent studies, children with DLD tend to present a greater number of spelling errors in words that depend on phonographemic conversion and greater ease in correctly spelling arbitrary spelling words, which are more linked to the lexical route and are less dependent on phonological skills^(11,12)^. Most of these studies were conducted in foreign languages with different characteristics of opacity and transparency^(11,12)^. However, the data from this study reinforce this hypothesis since we observed the same error profile in a transparent language, such as Brazilian Portuguese (BP), although it is common for orthographic writing to occur later due to the multiple representations that BP presents in the sense of the phoneme for the grapheme^(13)^. The need for further studies in the area is highlighted to advance the understanding of the process of acquisition of writing by DLD children literate in BP.

About decoding, the literature^(3)^ points out that children with language disorders tend to have difficulties related to understanding oral language and decoding ability, which is reinforced in the data of the present study, as children from the RG had a slower decoding time and a higher percentage of errors than their typical peers.

This study also points out the length of the word as an important variable, since there was an increase in the decoding time as a function of the length of the words for both groups, being always higher in the RG. Regarding pseudowords, which are more related to the phonological route, there was an increase in time for both groups, which was more expressive, once again, in RG. Therefore, the alterations verified in the decoding abilities of children with DLD in this study reinforce the hypothesis that the phonological processing difficulties present in this population, described in different studies^(3-5)^, and which were not the object of this study, may interfere with their literacy process. In this way, it is suggested, in future research, further investigation on these aspects.

When the individual uses the phonological route to read, the decoding time tends to be longer and the decoding is less fluent^(4)^. As mentioned, children with DLD have difficulties in phonological processing, which can also influence their ability to recognize words when they are learning to read. In addition, the difficulty in the phonological aspect of these children reduces the efficiency of metaphonological skills, such as phonological awareness and phonological short-term memory, important for decoding, and tend to be more related to their linguistic age than chronological^(2,4)^.

Even though this theme is addressed in greater depth in the world^(3,6,7,12,13)^, there are still few studies of this nature in Brazil. Therefore, this study shows fundamental evidence regarding the performance of children with DLD in decoding and writing skills and reinforces the importance of further studies in the area.

## CONCLUSION

Data show that children with DLD tend to have longer decoding times than their typical peers and below-expected results in writing, similar to what we observe in world literature. Furthermore, a longer decoding time and a lower percentage of correct answers were observed regardless of the length of the word, with greater difficulty in pseudowords. This study enables reflections on the written language performance of DLD children and reinforces the need for studies in the area.
