# Supplementary material for: Performance in decoding and writing of children with Developmental Language Disorder: preliminary data
Source: Codas. 2023 Oct 23;36(1):e20220318. doi: 10.1590/2317-1782/20232022318en (PMC10715580; doi:10.1590/2317-1782/20232022318en)
Supplement: Translation

# Translation: Desempenho em decodificação e escrita de crianças com Transtorno do Desenvolvimento da Linguagem: dados preliminares

**DOI:** 10.1590/2317-1782/20232022318pt

**Authors:** Aparecido José Couto Soares, Gabriele Hilário Cardoso Santos, Débora Maria Befi-Lopes

**Affiliations:** 1 Departamento de Fonoaudiologia da Universidade Federal de São Paulo - UNIFESP - São Paulo (SP), Brasil.; 2 Departamento de Fisioterapia, Fonoaudiologia e Terapia Ocupacional, Faculdade de Medicina, Universidade de São Paulo - USP - São Paulo (SP), Brasil.

**Keywords:** Linguagem, Transtorno Específico de Linguagem, Leitura, Aprendizagem, Avaliação

## Abstract

**Objetivo:**

Verificar o desempenho de crianças com diagnóstico de Transtorno do Desenvolvimento da Linguagem em provas de decodificação e escrita para assim entender melhor suas manifestações e o processo de aquisição das habilidades da linguagem escrita.

**Método:**

Foram sujeitos do estudo 80 crianças. Compuseram o Grupo-Pesquisa 16 crianças com diagnóstico de Transtorno do Desenvolvimento da Linguagem, sendo 13 do gênero masculino e 3 do gênero feminino, média de idade de 7,3. O Grupo Controle contou com 64 sujeitos pareados em gênero, idade, escolaridade e nível sócio econômico com o Grupo Controle na proporção 4:1. Foi realizada avaliação da habilidade de decodificação de palavras e pseudopalavras de ambos os grupos, contabilizando-se o tempo de leitura de palavras corretas e a porcentagem de acertos, considerando-se também a extensão da palavra/pseudopalavra. A avaliação da escrita foi realizada no grupo controle, que teve seus erros ortográficos analisados e categorizados. Todos os dados passaram por análise estatística descritiva e inferencial.

**Resultados:**

Os dados indicaram maior tempo de decodificação e menor porcentagem de acertos para as crianças do Grupo Pesquisa. Em relação aos erros ortográficos, observou-se a predominância de erros de ortografia arbitrária.

**Conclusão:**

Os dados evidenciam que crianças com Transtorno do Desenvolvimento da Linguagem tendem a apresentar maior tempo de decodificação, maior porcentagem de erros do que seus além, de erros ortográficos mais concentrados na ortografia natural.

## INTRODUÇÃO

O transtorno do desenvolvimento da linguagem (TDL) caracteriza-se pela presença de alterações significativas no processo de aquisição e desenvolvimento da linguagem, excluindo-se crianças com alterações justificadas por fatores sócio-ambientais, bi ou multilinguismo além de condições biomédicas onde são esperadas alterações de linguagem^(1,2)^.

Comumente observa-se nessas crianças prejuízos na aquisição lexical, que varia conforme o grau do transtorno e a criança pode apresentar dificuldades no vocabulário expressivo e receptivo. Ademais, os prejuízos nos aspectos fonológicos é uma característica importante, sendo comum essas crianças apresentarem fala ininteligível. Alguns estudos^(3,4)^ apontam déficits importantes nos componentes do processamento fonológico em crianças com TDL, como a memória de curto prazo fonológica e a consciência fonológica, que são consideradas habilidades fundamentais para o processo de aquisição da linguagem em sua modalidade escrita^(3)^.

Neste contexto, sabe-se que as habilidades do processamento fonológico associam-se com o sucesso no processo de aprendizagem porque somadas são responsáveis respectivamente, pela capacidade de análise da estrutura sonora da fala, retenção de informações e o acesso rápido a representações das informações fonológicas da língua^(5)^. Todavia, conforme citado, essas são habilidades prejudicadas em crianças com TDL devido às alterações em diversos subsistemas da linguagem^(1)^.

Essas alterações, portanto, acarretam em um tempo maior para a solidificação linguística em crianças com TDL quando comparadas à população em desenvolvimento típico e levam à repercussões importantes no processo de aquisição da linguagem escrita^(4)^. Sabe-se que essas crianças apresentam dificuldades referentes à leitura e à escrita, provavelmente decorrentes dos prejuízos da linguagem oral e do processamento fonológico característicos do TDL^(6)^. Há um crescente no número de estudos relacionados às habilidades de linguagem escrita de crianças com TDL, bem como a importância da sua caracterização para a efetuação de diagnósticos diferenciais em relação aos Transtornos Específicos de Aprendizagem e, até mesmo, a co-ocorrência destes quadros^(7)^.

Apesar disso, são escassos os estudos brasileiros que se dedicam a melhor entender as alterações de linguagem escrita dessas crianças, bem como seu processo de aquisição. Isso decorre do fato de que até a mudança de paradigma e de nomenclatura de Distúrbio Específico de Linguagem (DEL) para Transtorno do Desenvolvimento de Linguagem^(1)^, as manifestações da linguagem escrita eram pouco investigadas nesta população, principalmente no Brasil. No entanto, evidencia-se a importância de uma abordagem holística das alterações de crianças TDL, principalmente considerando-se que tal transtorno acomete, longitudinalmente, todos os subsistemas da linguagem^(4)^, que levarão a diferentes impactos ao longo da vida do indivíduo e, consequentemente, na sua trajetória escolar.

Dessa forma, torna-se primordial estudos que investiguem as manifestações na linguagem escrita de crianças com TDL, para melhor entender os prejuízos desta população e delinear não apenas planejamentos terapêuticos que envolvam a linguagem escrita, mas desenvolver ações que promovam o desenvolvimento de políticas públicas educacionais para essas crianças que, atualmente, não são amparadas por nenhuma legislação a respeito. Como hipóteses, espera-se que as crianças com TDL apresentem prejuízos na decodificação e na aquisição da escrita.

Sendo assim, o objetivo do presente estudo é verificar o desempenho de crianças com diagnóstico de TDL em provas de decodificação e escrita sob ditado.

## MÉTODO

Estudo retrospectivo transversal, aprovado pelo Comitê de Ética e Pesquisa da Faculdade de Medicina da Universidade de São Paulo sob nº 2.262.300. O estudo foi realizado no Laboratório de Investigação Fonoaudiológica em Pediatria do curso de Fonoaudiologia da Universidade de São Paulo. Em decorrência de a pesquisa ser retrospectiva, realizada em banco de dados, o Termo de Consentimento Livre e Esclarecido foi dispensado.

Participaram deste estudo 80 crianças na faixa etária de 6 a 10 anos de idade, separadas em dois grupos. O Grupo-Pesquisa (GP) foi composto por 16 crianças, sendo 13 do sexo masculino e 3 do sexo feminino, média de idade de 7,3, com diagnóstico de Transtorno do Desenvolvimento da Linguagem (TDL) baseados em critérios internacionais recentes^(1,2)^, e que foram atendidas no ambulatório do referido Laboratório. O público do serviço é prioritariamente de nível socioeconômico médio-baixo. Importante ressaltar que tal variável foi considerada quando da avaliação e diagnóstico de TDL, conforme diretrizes mais recentes, que incluem uma bateria de avaliação de todos os subsistemas da linguagem e suas habilidades subjacentes^(1,2)^. Os critérios de inclusão para este grupo foram: possuir diagnóstico de TDL, estar em idade de escolarização formal (6 a 10 anos de idade) e estar regularmente matriculado no Ensino Fundamental I. O Grupo Controle (GC) contou com 64 sujeitos pareados em gênero, idade, escolaridade e nível socioeconômico com o GP na proporção 4:1, ou seja, cada criança do GP, foi pareada com 4 crianças do GC.

O GC foi construído especificamente para a prova de decodificação e conta com crianças com desenvolvimento de linguagem oral, leitura e escrita típicos, confirmado por procedimentos fonoaudiológicos realizados em estudo anterior^(8)^. A média de idade do GC foi de 7,2. Os critérios de inclusão do GC foram: não possuir queixas ou alterações de linguagem oral e escrita; estar regularmente matriculado em escola do ensino fundamental I; não possuir queixas de aprendizagem; possuir desempenho adequado em triagem fonoaudiológica realizada no estudo anterior^(8)^. Os indivíduos do GP foram avaliados quanto às suas habilidades de decodificação e escrita e as crianças do GC apenas quanto à decodificação. Isso decorre do fato de a prova de decodificação utilizada ter parâmetros e variáveis específicas que foram publicadas recentemente^(8)^ e a análise da escrita ser realizada por instrumento publicado em forma de teste padronizado^(9)^, dispensando a necessidade de Grupo Controle para tal variável.

Para avaliação e análise da decodificação foi utilizado o Protocolo de Acompanhamento do Desenvolvimento da Decodificação (PRADE)^(8)^ que consiste de palavras balanceadas linguisticamente conforme as regras de decodificação do Português Brasileiro (PB) respeitando-se também a variação de extensão de palavras de mono a polissílabos para crianças desta faixa escolar. A prova também conta com não-palavras que foram derivadas das palavras reais e que também seguem as regras de decodificação do PB, bem como a variação de mono a polissílabos. Ambas as tarefas foram realizadas de forma presencial e consistiram em solicitar à criança a decodificar as palavras da maneira que elas acreditavam ser a forma correta. As palavras são apresentadas iniciando-se pelos monossílabos, seguindo para dissílabos e assim sucessivamente. Quando a criança comete dez erros consecutivos a prova é finalizada. O procedimento é o mesmo para palavras e não-palavras. Foram contabilizados o tempo de decodificação de palavras corretas e a porcentagem de acertos tanto para cada tipo de estímulo (de mono a polissílabo) quanto valores totais para a categoria (palavras ou não palavras). A escolha de tal instrumento se deu em decorrência de este analisar especificamente a decodificação, que sabe-se ser habilidade fundamental para as fases posteriores da alfabetização e por conter estímulos que são baseados na estrutura do Português Brasileiro, com adequado balanceamento linguístico, conforme descrito anteriormente. Os dados foram tabulados em planilha específica e passaram por análise estatística.

Em relação à escrita, foi utilizada uma lista de palavras utilizada em estudo anterior, que mostraram-se adequadas para crianças com TDL^(10)^. A prova consiste em um ditado de oito palavras e oito pseudopalavras; ambas as listas de palavras foram compostas de palavras dissílabas com estrutura CV (consoante-vogal); além disso, as palavras possuem correspondência fono-grafêmica consideradas transparentes. Realizou-se análise do desempenho em escrita e, para crianças com escrita alfabética, foi realizada categorização do perfil de erros ortográficos com base nas diretrizes do Teste Pró-Ortografia^(9)^. Nesta perspectiva, os erros foram classificados, em porcentagem, em erros de ortografia natural e arbitrária^(9)^. Os dados foram tabulados em planilha específica e passaram por análise estatística.

Foi realizada análise estatística com o objetivo de caracterizar os grupos em relação ao tempo de leitura e porcentagem de acertos de acordo com o tipo e a extensão das palavras, além dos valores totais; investigou-se também o efeito do grupo, do tipo de palavra e da extensão da palavra sobre o tempo e a porcentagem de acertos. O valor de significância estatística adotado foi igual a 5% (p ≤ 0,05). Utilizou-se o software SPSS Statistics, versão 28.0. Foram realizadas também equações de estimações generalizadas (GEE) para verificar os efeitos para cada variável isoladamente, intragrupo e também os efeitos da interação entre todas as variáveis estudadas (tipo de palavra, extensão e valores totais) intergrupo. Em relação à escrita, foi verificada a porcentagem de crianças com nível alfabético de escrita e, destas, a porcentagem da tipologia de erros, de ortografia natural ou arbitrária.

## RESULTADOS

No que diz respeito à análise da escrita, das crianças classificadas em nível alfabético, os dados indicaram que, em média, 76,58% dos erros foram de ortografia natural e 35,13% de ortografia arbitrária. Observou-se também grande variabilidade no desvio padrão com valores de 22,95 e 18,68, respectivamente.

Em relação a decodificação, observou-se maior tempo de decodificação no GP em comparação com o GC (Tabela 1). Além disso, a extensão da palavra foi uma variável importante e levou ao aumento do tempo de leitura em ambos os grupos, mas em maior proporção no GP. Tal fato foi observado tanto na decodificação de palavras quanto em pseudopalavras (Tabela1). Em relação à porcentagem de acertos tanto das palavras quanto das pseudopalavras, o desempenho do GP foi consideravelmente menor quando comparado ao GC (Tabela 1).

**Tabela 1:** Valores descritivos do tempo de leitura (em segundos) e porcentagem de acertos de acordo com o tipo de palavra, a extensão da palavra e o grupo

**TEMPO DE LEITURA**										
**Tipo de palavra**	**Extensão da palavra**	**Grupo**	**n**	**Média**	**DP**	**IC 95%**		**Mediana**	**Mín.**	**Máx.**
						**LI**	**LS**			
Palavra	Monossílabo	GC	54	7,09	2,56	6,43	7,72	7,10	1,20	14,70
		GP	16	19,56	7,59	16,50	23,12	18,00	11,00	37,00
	Dissílabo	GC	52	23,86	10,11	21,28	26,54	22,10	1,10	51,30
		GP	14	72,71	42,67	55,70	95,00	66,00	29,00	201,00
	Trissílabo	GC	50	32,26	14,42	28,17	36,39	32,45	5,50	66,20
		GP	9	109,89	36,08	88,00	132,00	106,00	61,00	183,00
	Polissílabo (4 sílabas)	GC	42	38,69	24,56	32,19	46,87	32,15	5,20	137,40
		GP	6	93,50	22,42	76,33	111,20	98,50	64,00	118,00
	Polissílabo (5 sílabas)	GC	41	22,00	8,59	19,55	24,66	20,10	5,70	39,50
		GP	5	58,00	22,52	43,40	72,60	57,00	39,00	94,00
	Total	GC	55	104,80	52,25	90,58	119,42	113,00	4,50	209,00
		GP	16	198,19	123,19	142,29	253,37	223,00	18,00	408,00
Pseudopalavra	Monossílabo	GC	45	8,93	2,85	8,23	9,64	8,50	2,10	19,50
		GP	9	22,78	12,47	16,67	30,44	17,00	12,00	52,00
	Dissílabo	GC	42	30,58	9,15	28,05	33,12	28,20	17,80	57,20
		GP	8	94,00	44,04	64,63	124,62	84,50	45,00	154,00
	Trissílabo	GC	45	42,28	14,88	37,82	46,16	41,80	7,60	85,30
		GP	6	129,67	34,94	105,33	157,54	138,50	88,00	179,00
	Polissílabo (4 sílabas)	GC	42	37,97	15,13	33,62	42,94	36,70	8,20	79,20
		GP	4	114,25	38,35	86,23	146,25	114,50	68,00	160,00
	Polissílabo (5 sílabas)	GC	39	24,42	9,15	21,75	27,02	24,20	6,80	46,20
		GP	4	65,75	17,21	54,25	78,56	63,00	49,00	88,00
	Total	GC	48	127,84	53,91	110,48	142,97	133,60	6,50	219,20
		GP	9	272,56	153,87	180,79	362,44	273,00	12,00	553,00
**PORCENTAGEM DE ACERTOS**										
**Tipo de palavra**	**Extensão da palavra**	**Grupo**	**n**	**Média**	**DP**	**IC 95%**		**Mediana**	**Mín.**	**Máx.**
						**LI**	**LS**			
Palavra	Monossílabo	GC	64	64,06	41,81	54,43	73,78	83,33	0,00	100
		GP	16	56,56	28,03	42,80	69,48	52,50	16,00	100
	Dissílabo	GC	64	55,18	40,24	45,63	64,82	75,00	0,00	100
		GP	16	49,26	31,47	33,02	64,52	49,98	0,00	93,75
	Trissílabo	GC	64	52,41	41,41	42,07	63,14	59,09	0,00	100
		GP	16	25,52	30,55	12,15	39,98	14,28	0,00	85,71
	Polissílabo (4 sílabas)	GC	64	50,10	42,12	39,85	60,54	62,50	0,00	100
		GP	16	16,80	28,93	3,91	32,03	0,00	0,00	81,25
	Polissílabo (5 sílabas)	GC	64	51,76	43,58	41,80	61,52	68,75	0,00	100
		GP	16	17,19	29,18	5,47	31,02	0,00	0,00	87,50
	Total	GC	64	51,94	39,81	42,55	61,73	65,71	0,00	97,14
		GP	16	25,37	30,45	10,72	40,61	13,50	0,00	86,19
Pseudopalavra	Monossílabo	GC	64	51,04	37,66	41,94	60,16	66,67	0,00	100
		GP	16	39,79	39,07	21,87	57,29	33,33	0,00	100
	Dissílabo	GC	64	51,56	40,72	42,29	60,84	62,50	0,00	100
		GP	16	30,98	34,02	15,82	46,61	19,75	0,00	100
	Trissílabo	GC	64	43,11	33,94	35,31	51,12	54,55	0,00	95,45
		GP	16	21,87	32,69	6,53	36,63	0,00	0,00	86,36
	Polissílabo (4 sílabas)	GC	64	33,30	29,86	25,98	40,71	37,50	0,00	87,50
		GP	16	16,02	32,19	1,17	32,42	0,00	0,00	93,75
	Polissílabo (5 sílabas)	GC	64	36,52	35,17	28,91	44,73	37,50	0,00	100
		GP	16	12,50	27,76	1,56	25,00	0,00	0,00	87,50
	Total	GC	64	41,50	32,89	34,05	49,27	52,14	0,00	92,86
		GP	16	23,09	29,36	10,14	37,00	13,54	0,00	88,52

Legenda: n = número de participantes; DP = Desvio padrão; Mín. = Mínimo; Máx. = Máximo; IC 95% = intervalo de confiança de 95% calculado com 1000 amostras *bootstrap*; LI = limite inferior; LS = limite superior

A Tabela 2 confirma os pontos analisados quanto à decodificação de forma conjunta, indicando diferença estatística nas múltiplas variáveis da presente pesquisa. Dessa forma, observa-se interação entre todas as variáveis, ou seja, todas elas influenciam o desempenho em decodificação, porém, o maior impacto foi observado no GP, verificando-se prejuízos em todas as variáveis estudadas (palavra/pseudopalavra, extensão da palavra/pseudopalavra, valores totais de tempo de decodificação e porcentagem de acertos).

**Tabela 2:** Teste de efeitos para cada variável e para a interação entre as variáveis das Equações de Estimações Generalizadas elaborados para tempo e porcentagem de acertos

**Variável**	**Par.**		**Efeitos**						
		**Intercepto**	**G**	**T**	**E**	**G x T**	**G x E**	**T x E**	**G x T x E**
Tempo	X^2^ de Wald	10396,960	159,466	29,301	791,129	2,357	10,365	5,758	6,574
	gl	1	1	1	4	1	4	4	4
	p	< 0,001*	**< 0,001** *	< 0,001*	< 0,001*	0,125	0,035*	0,218	0,160
Acertos (%)	X^2^ de Wald	269,657	4,320	14,827	26,053	2,880	11,609	2,076	1,229
	gl	1	1	1	4	1	4	4	4
	p	< 0,001*	0,038*	< 0,001*	< 0,001*	0,090	0,021*	0,722	0,873

Teste X^2^ de Wald

* Valor estatisticamente significante no nível de 5% (p ≤ 0,05)

Legenda: Par. = Paraâmetro; gl = graus de liberdade; G = Grupo; T = Tipo de palavra; E = Extensão da palavra

## DISCUSSÃO

Este estudo teve como objetivo verificar o desempenho de crianças com diagnóstico de TDL em provas de decodificação e escrita para assim entender melhor suas manifestações e o processo de aquisição das habilidades da linguagem escrita nesta população. A base da aquisição da linguagem escrita advém de habilidades adquiridas e aprimoradas a partir da linguagem oral^(11)^, o que em crianças com transtorno do desenvolvimento da linguagem ocorre de modo mais lento^(4)^.

Neste contexto, considerando as crianças em nível alfabético de escrita notou-se uma porcentagem maior de erros de escrita natural do que arbitrários, o que corrobora a hipótese de que as alterações em processamento fonológico dessas crianças dificultam a conversão fonema-grafema e consequentemente o desenvolvimento da escrita^(11)^.

É importante salientar que em estudos recentes, crianças com TDL tendem a apresentar maior número de erros ortográficos em palavras que dependem da conversão fono-grafêmica e maior facilidade em grafar corretamente palavras de ortografia arbitrária, que estão mais ligadas à rota lexical e são menos dependentes de habilidades fonológicas^(11,12)^. Ressalta-se, também que a maioria destes estudos foram realizados em línguas estrangeiras com diferentes características de opacidade e transparência^(11,12)^. Entretanto, os dados do presente estudo reforçam tal hipótese uma vez que observamos o mesmo perfil de erros em uma língua transparente, como é o Português Brasileiro (PB), apesar de ser comum a escrita ortográfica ocorrer mais tardiamente devido às múltiplas representações que o PB apresenta no sentido do fonema para o grafema^(13)^. Salienta-se a necessidade de mais estudos na área para que se avance no entendimento do processo de aquisição da escrita por crianças TDL alfabetizadas em PB.

No que se refere à decodificação, a literatura^(3)^, aponta que crianças com distúrbios da linguagem tendem a apresentar dificuldades relacionadas desde a compreensão da linguagem oral até a capacidade de decodificação, o que é reforçado nos dados do presente estudo, pois as crianças do GP apresentaram tempo de decodificação mais lento e maior porcentagem de erros em relação a seus pares típicos.

O presente estudo também aponta a extensão da palavra como uma variável importante, uma vez que houve aumento no tempo de decodificação em função da extensão das palavras para ambos os grupos, sendo sempre superior no GP. Em relação às pseudopalavras, que estão mais relacionadas à rota fonológica, verificou-se aumento no tempo de ambos os grupos sendo este mais expressivo, uma vez mais, no GP. Sendo assim, as alterações verificadas nas habilidades de decodificação das crianças com TDL deste estudo reforçam a hipótese de que as dificuldades de processamento fonológico presente nesta população descritas em diferentes pesquisas^(3-5)^, e que não foram objeto do presente estudo, podem interferir em seu processo de alfabetização. Dessa maneira, sugere-se, em pesquisas futuras, investigação mais aprofundada sobre tais aspectos.

Sabe-se que, quando o indivíduo se utiliza da rota fonológica para ler, o tempo de decodificação tende a ser maior e a decodificação apresenta-se menos fluente^(4)^. Conforme mencionado, as crianças com TDL apresentam dificuldades no processamento fonológico, o que pode influenciar também em sua capacidade de reconhecimento das palavras quando estão aprendendo a ler. Além disso, a dificuldade no aspecto fonológico dessas crianças reduz a eficiência das habilidades metafonológicas, como a consciência fonológica e memória de curto prazo fonológica, importantes para a decodificação, e que tendem a estar mais relacionadas à sua idade linguística do que cronológica^(2,4)^.

Cabe salientar que, apesar de tal temática ser abordada mais aprofundadamente no mundo^(3,6,7,12,13)^ ainda há poucos estudos desta natureza no Brasil. Sendo assim, o presente estudo apresenta evidências fundamentais a respeito do desempenho de crianças com TDL em habilidades de decodificação e escrita e reforça a importância de novos estudos na área.

## CONCLUSÃO

Os dados evidenciam que crianças com TDL tendem a apresentar maior tempo de decodificação do que seus pares típicos e resultados abaixo do esperado no que se refere à escrita, consonante com o que se observa na literatura mundial. Ademais, observou-se maior tempo de decodificação e menor porcentagem de acertos independentemente da extensão da palavra, com maior dificuldade nas pseudopalavras. O presente estudo possibilita reflexões sobre o desempenho em linguagem escrita de crianças TDL e reforça a necessidade de estudos na área
